# Bibliography

**Published:** 1897-01

**Authors:** 


### Bibliography.

Compend of Dental Pathology and Therapeutics. By Henry H. Burchard, M.D., D.D.S. Special Lecturer upon Dental Pathology and Therapeutics, Philadelphia Dental College. Philadelphia: The S. S. White Dental Manufacturing Company, 1896.

It is, perhaps, unreasonable to expect that compends will ever cease to be issued while the demand exists for a short road to knowledge, and, while it is to be regretted that such a demand does exist, it is a satisfaction to find that this class of books shows a continued improvement.

The present volume is not prepared, as the author states in his preface, "as an aid to students in memorizing answers for an examination," and yet it is feared it will have exactly that result. The author has "prepared it primarily for students attending his own lectures," and it is hoped that the generally clear definitions of pathological conditions will have a beneficial result.

Books of this character defy criticism, as they give the briefest outline of the subjects treated,—in fact, in many instances tend to lead the student astray. The author has, in this effort, succeeded very well in condensing facts, as he understands them, and has evidently tried, and with excellent success, to give a consensus of the various opinions held by writers upon the pathology of the oral cavity; but the natural result of a question and a brief answer is a tendency to dogmatism, and this is frequently apparent throughout the pages of the book. Its greatest defect, common to all such books, is the effort to condense into a few lines that which should require pages of description.

It seems to the writer that it is a mistake for a teacher to issue such a book for his students. The experience has been of all those long in the service that such compends invariably end in becoming a plague to the author, and, it is surmised, this will be no exception.

The book can be recommended as one of the best of its class, and its contents will not only be appreciated by students, Boards of Examiners, but all that class who aim at a superficial knowledge of dental pathology.

THÉRAPEUTIQUE DE LA BOUCHE ET DES DENTS, HYGIÈNE BUCCALE ET ANESTHÉSIE DENTAIRE. Par le Dr. Maurice Roy, Dentiste des Hôpitaux de Paris, etc. Librairie J. B. Baillière et Fils. Paris, 1897.

This is a compend of dental therapeutics, and has similar uses for French students as that heretofore described under Dr. Burchard's book. It combines a description of all remedial agents used in dentistry and the method of treatment, the administration of anæsthetics, with the necessary precautions to be taken, contra-indications in administration, and the care necessary in case of serious results. The author very properly gives credit for the discovery of anæsthesia to Horace Wells, and the use of ether to Jackson and Morton.

The book, small as it is, contains a variety of information, and becomes, in this sense, worthy a place among books of reference. Its defects are common to all compends.

